# Signed, Sealed, Delivered: Microenvironmental Modulation of Extracellular Vesicle-Dependent Immunoregulation in the Lung

**DOI:** 10.3389/fcell.2016.00094

**Published:** 2016-08-30

**Authors:** Daniel J. Schneider, Jennifer M. Speth, Marc Peters-Golden

**Affiliations:** ^1^Division of Pulmonary and Critical Care Medicine, University of Michigan Medical SchoolUSA; ^2^Graduate Program in Immunology, University of Michigan Medical SchoolUSA

**Keywords:** lung, vesicle, microvesicle, exosome, SOCS, alveolar, macrophage, epithelial

## Abstract

Unconventional secretion and subsequent uptake of molecular cargo via extracellular vesicles (EVs) is an important mechanism by which cells can exert paracrine effects. While this phenomenon has been widely characterized in the context of their ability to promote inflammation, less is known about the ability of EVs to transfer immunosuppressive cargo. Maintenance of normal physiology in the lung requires suppression of potentially damaging inflammatory responses to the myriad of insults to which it is continually exposed. Recently, our laboratory has reported the ability of alveolar macrophages (AMs) to secrete suppressors of cytokine signaling (SOCS) proteins within microvesicles (MVs) and exosomes (Exos). Uptake of these EVs by alveolar epithelial cells (AECs) resulted in inhibition of pro-inflammatory STAT activation in response to cytokines. Moreover, AM packaging of SOCS within EVs could be rapidly tuned in response to exogenous or AEC-derived substances. In this article we will highlight gaps in knowledge regarding microenvironmental modulation of cargo packaging and utilization as well as EV secretion and uptake. Advances in these areas are critical for improving understanding of intercellular communication in the immune system and for therapeutic application of artificial vesicles aimed at treatment of diseases characterized by dysregulated inflammation.

## Introduction

**Figure 1:**
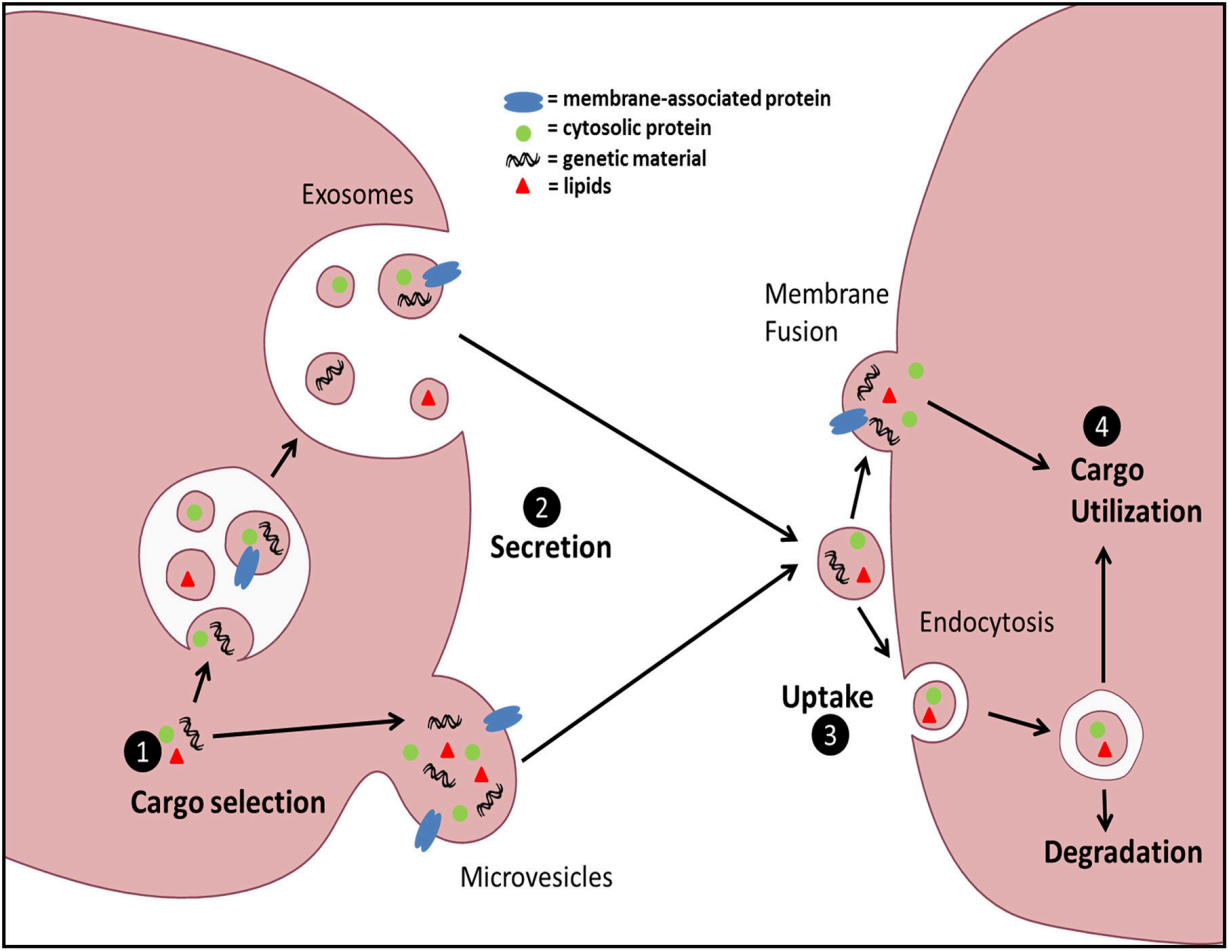
**General schematic of EV biogenesis and uptake**. EV cargo can be sorted into two distinct EV populations: Exos or MVs. Exos are generated via inward budding of the multivesicular body (MVB) membrane and subsequently released via fusion of MVBs with the plasma membrane. MVs are generated by direct outward budding and fission of the plasma membrane. Uptake of EVs can be accomplished by either direct membrane fusion and release of EV contents into the target cell cytoplasm, or through various forms of endocytosis. Finally, the fate of the intravesicular cargo can be influenced by the form of uptake and the ability of the contents to avoid lysosomal degradation.

Intercellular communication is vital for multicellular organisms to respond and adapt to changes in the environment. It is a cornerstone of the immune response and is classically accomplished via either direct cell-cell contact or secretion of soluble mediators. Extracellular vesicles (EVs) have emerged recently as additional vectors of communication and represent an area of intense investigation. EVs are small membrane-delimited packets which, by transferring diverse forms of biologically active cargo (lipids, RNA, DNA, soluble and surface proteins) from donor to recipient cells, participate in both homeostasis and disease. EVs comprise a spectrum of structures that vary in size, membranes of origin, mechanisms of release, and surface and internal cargo. The best characterized subsets of these are microvesicles (MVs) and exosomes (Exos). MVs originate by direct plasma membrane budding and are ~100–1000 nm in diameter, while Exos originate from endosomal membranes within multivesicular bodies and are ~30–150 nm in diameter. A number of steps in vesicle trafficking ultimately dictate transduction of information (Figure 1); these include cargo sorting into vesicles and vesicle formation and release from source cells, as well as vesicle uptake and cargo utilization within target cells. Fundamental mechanisms governing these processes under normal conditions are beginning to be elucidated, yet how these steps are modulated by external or microenvironmental cues remains poorly understood, and is a focus of this review.

Comprising an enormous direct interface with the outside environment, the alveolar surface of the lung is uniquely exposed to diverse challenges including microbes, pollutants, and antigens. Preserving the delicate alveolar epithelial barrier to ensure maintenance of gas exchange in the face of this continual and dynamic onslaught requires a tightly-regulated host immune response. The key cellular players responsible for immune and inflammatory responses at this interface are the alveolar epithelial cells (AECs)—which comprise the surface—and alveolar macrophages (AMs)—the resident immune cell of the distal lung. We have recently identified a novel means for maintaining immune homeostasis at this site in which inflammatory signaling within AECs is restrained by AM-derived EVs. We were interested to find that various aspects of vesicular cargo trafficking can additionally be modulated by distinct environmental cues. In this brief review, we place this concept of microenvironmental influence on EV communication in a broader context of immune function. We then discuss the implications of these findings in directing future studies on translational aspects of vesicles in diagnosis and treatment of disease.

## Extracellular vesicles in immune function

Although EVs have been identified in virtually all organ systems, they have been most extensively studied in vascular and cancer biology (reviewed in Desrochers et al., 2016; Osteikoetxea et al., 2016). However, a growing body of recent literature has implicated EVs in the pathogenesis and—to a lesser extent—the resolution of inflammation (Norling and Dalli, 2013; Fernández-Messina et al., 2015), and have shown convincing evidence of functionally relevant EV-mediated delivery to target cells of cargo including lipids (Tang et al., 2010), mRNA (Valadi et al., 2007), and miRNA (Pegtel et al., 2010). Very little is known about the role of EVs in lung physiology. Recently, our lab has discovered a novel mechanism by which AMs can modulate host response through immunosuppressive EVs.

## Transcellular modulation of immune function in the alveolar microenvironment

**Figure 2:**
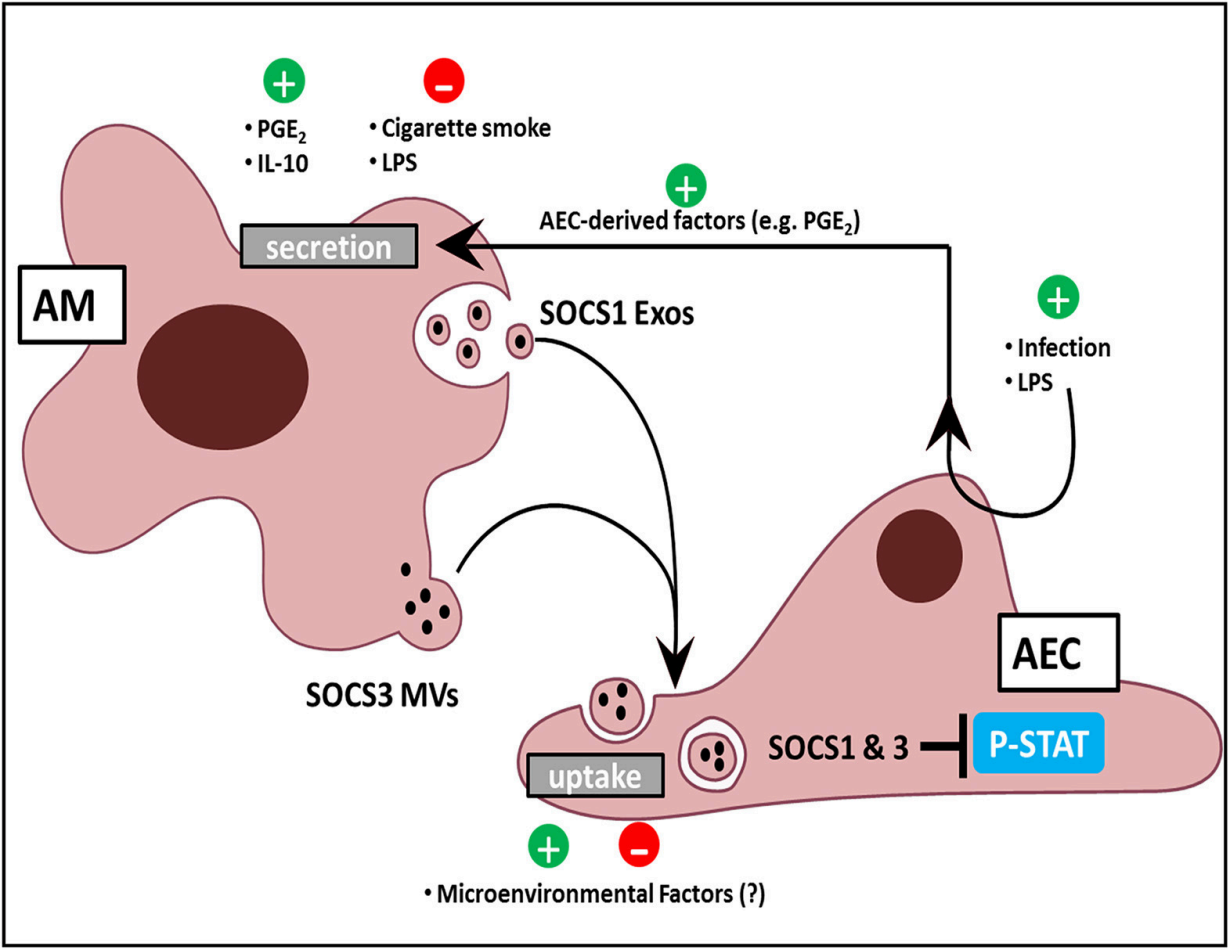
**AM and AEC crosstalk in the lung**. AMs release Exos and MVs containing SOCS1 and SOCS3, respectively. Positive regulators of SOCS release include the bioactive lipid PGE_2_, the cytokine IL-10, and AEC-derived mediators in response to LPS and infection. Negative regulators include acute, direct AM exposure of LPS, and cigarette smoke. AEC uptake of SOCS-containing EVs might likewise be modulated by constituents of the normal alveolar milieu or by exogenous factors to which the lung is exposed. Additionally, EV uptake by AECs results in inhibition of cytokine-induced STAT activation.

Phosphorylation and activation of the STAT family of transcription factors by JAK kinases is integral to transduction of inflammatory signals in response to cytokines. STAT-dependent transcription of inflammatory genes such as chemokines by AECs plays an important role in leukocyte recruitment to the alveolar space. The endogenous brake on this pathway is the family of SOCS proteins, which can directly inhibit STAT activation. Although SOCS proteins had never been identified previously in the extracellular space, we found that AMs constitutively secrete two distinct SOCS proteins (SOCS1 and SOCS3) within EVs (Bourdonnay et al., 2015). Furthermore, considering the aforementioned importance of restraining inflammation in the lung, we were surprised to find that AECs express very little endogenous SOCS3 protein, although this is consistent with immunohistochemical analysis of normal lung (Akram et al., 2014). This led us to hypothesize that AM release of EV-contained SOCS proteins may serve as an “external” source of SOCS for AECs during inflammatory responses. Both conditioned medium (CM) from AMs as well as EVs purified from AM CM possessed the ability to inhibit cytokine-induced STAT activation and expression of a STAT-dependent gene product, MCP-1 (or CCL-2) in AECs *in vitro* and in the lung *in vivo*. This inhibitory effect in AECs was eliminated when either SOCS1 or SOCS3 expression was silenced in the source AMs. Lung lavage fluid from healthy smoking humans, or from mice subjected to smoke exposure for several days, showed reduced SOCS levels within MVs. These results, summarized in Figure 2, suggest that constitutive elaboration of SOCS proteins by AMs restrains inflammatory signaling in the alveolar epithelium, but that this process is impaired during smoking. We speculate that this “brake malfunction” facilitates inflammatory responses to smoking.

## Cargo selection

In order for EVs to be meaningful regulators of the immune response, their cargo should be subject to dynamic selection in response to microenvironmental cues. Although EV cargo is dictated by the intracellular contents of its source cell, differences in profiles implying selective cargo packaging into vesicles are well-recognized. Since proteins which lack N-terminal signal sequences—such as SOCS proteins—cannot be secreted via a classical endoplasmic reticulum-Golgi pathway, it is not surprising that these are preferentially secreted within vesicles (Nickel and Rabouille, 2009). Additionally, ubiquitination of specific proteins actively targets them to endosomal sorting complexes required for transport into vesicles (Hanson and Cashikar, 2012). However, preferential packaging of cargo within distinct vesicle populations is less well-appreciated. Surprisingly, differential ultracentrifugation to separate these two EV subpopulations revealed that SOCS1 was primarily packaged within smaller Exos and SOCS3 within larger MVs (Figure 2). Exclusion of these fractions from AM CM abrogated their inhibitory effect on STAT activation in response to cytokines (Bourdonnay et al., 2015). Given the demonstrated effects of EVs on immunomodulation and the concept that the immune response is dictated by changes in the environment, especially applicable to the lung, it becomes essential to understand how these changes alter vesicular cargo trafficking.

We also found that brief (15–60 min) direct exposure of AMs to the classic inflammatory stimulus LPS inhibited SOCS secretion, while the anti-inflammatory mediators IL-10 and prostaglandin E_2_ (PGE_2_) increased SOCS secretion. The rapidity of these effects argues that transcriptional mechanisms were not operative. Interestingly, these effects also occurred in the absence of any change in the numbers of EVs secreted, indicating that these substances alter the packaging of SOCS proteins within the EVs (Bourdonnay et al., 2015; Figure 2). Since little is known of the molecular mechanisms by which cytosolic proteins such as SOCS 1 and 3 are selectively packaged into EVs, how environmental factors further shape their sorting remains unexplained. The capacity for SOCS packaging within EVs to be rapidly increased by mediators also suggested a possible means by which AECs could “instruct” AMs to secrete additional SOCS if needed. Indeed, AMs exposed to CM isolated from AECs pretreated for 24 h by LPS exhibited enhanced SOCS3 secretion (Speth et al., 2016). This indirect enhancement of AM SOCS3 secretion in response to LPS-stimulated AEC-derived factors was in stark contrast to the direct inhibitory effect of acute (1–2 h) LPS exposure on AMs. As AECs produced high levels of PGE_2_ in response to LPS treatment, we employed both pharmacologic and genetic inhibition of PGE_2_ synthesis to demonstrate that PGE_2_ elaboration by AECs served as the “request signal” for enhanced SOCS3 secretion by AMs (Speth et al., 2016; Figure 2). This form of EV-mediated crosstalk represents a way in which AMs can dynamically respond to microenvironmental cues to constrain endogenous inflammation in AECs during an innate immune response.

## EV secretion

There are many reported examples in which the number of released EVs is regulated by cues external to the source cell. LPS stimulation of dendritic cells (DCs) increases the number and changes the composition of MVs released (Obregon et al., 2006; Nolte-'t Hoen et al., 2013). Exos were released from DCs following T cell interaction with peptide-loaded MHC class II on DCs (Buschow et al., 2009). T cells also secrete Exos upon CD3 T cell receptor cross-linking (Blanchard et al., 2002). Mast cells release EVs in response to changes in Ca^++^ concentration in association with the process of degranulation (Raposo et al., 1997). Even though we observed no change in the numbers of SOCS-containing EVs in response to the stimuli discussed above, AM adherence to plastic, which is well known to trigger Ca^++^ influx and cellular activation, did result in increased EV secretion. ESCRT complexes, tetraspannins, membrane lectins, heat shock proteins, membrane curvature, and surface proteins are all reported to play a role in secretion of vesicles (Yáñez-Mo et al., 2015) and thus may mediate some of these changes in vesiculation in response to external stimuli.

## EV uptake

Information transfer via EVs requires that they either be internalized by, or trigger a plasma membrane-based response in, the target cells. Various forms of endocytosis represent the predominant internalization pathway reported for both MVs and Exos in target cells, with lesser roles reported for membrane fusion, micropinocytosis, and phagocytosis (reviewed in Mulcahy et al., 2014). In contrast to mechanisms governing vesicle formation and secretion, a clear divergence between uptake mechanisms for MVs and Exos has not been established. However, uptake of EVs is subject to physiologic regulation. As an example, vasopressin enhances uptake of EVs in the renal collecting system (Oosthuyzen et al., 2016). Transformed tumor cells have heightened uptake efficiency when compared to their pre-transformed counterparts (Nakase et al., 2015). The acidic pH of the tumor microenvironment has been suggested to facilitate membrane fusion of vesicles with recipient cells (Parolini et al., 2009). Secreted lipases from platelets in the context of inflammation enhance uptake of MVs into neutrophils (Duchez et al., 2015), although the mechanism for this effect remains uncertain. Our lab is currently investigating the impact of normal and pathologic extracellular constituents on uptake of MVs within AECs. Thus, like cargo selection and secretion of vesicles, uptake represents an additional step in vesicle trafficking that can be modulated by relevant microenvironmental substances.

## Functional utilization of EV cargo

The question of how cargo within internalized EVs ultimately reaches the compartments within recipient cells in which it can be functionally active remains enigmatic. This challenge is vividly illustrated by the case of SOCS proteins transferred in AM-derived EVs, which must reach the cytosol of recipient AECs in order to effectively inhibit STAT phosphorylation. One might imagine that membrane-membrane fusion would be the ideal uptake mechanism to enable cargo to circumvent the lysosomal degradation pathway and be delivered into the cytosol. But, as noted above, most reports favor endocytosis over membrane-membrane fusion as the operative mechanism in uptake. Investigators have endeavored to enhance the efficiency of membrane fusion by engineering EVs containing specific surface proteins. However, such modifications have been reported to both increase (Temchura et al., 2008) and decrease (Maguire et al., 2012) the delivery of functionally active cargo in recipient cells. Thus, engineering of vesicles for optimal delivery of functional cargo—important for their potential therapeutic applicability—depends on an understanding of factors facilitating the ultimate utilization of cargo.

## EV signaling with infectious challenges

Infections pose the most common challenge to the immune system, particularly in the lung. We have also investigated the effects of lung infection on SOCS packaging within AM-derived vesicles. In contrast to what we observed in cigarette smoking, the lungs of mice subjected to both viral and bacterial infection had increased levels of SOCS3 within MVs, in the absence of any change in MV number (Speth et al., 2016). By contrast, no change in SOCS1 levels within Exos was seen. We speculate that differential regulation of vesicular packaging of these two SOCS family members may reflect an attempt to restrain excessive pathologic inflammation (accomplished by increasing SOCS3) without compromising protective STAT-1-mediated antimicrobial defense (which would be inhibited by SOCS1).

That viral particles and host-derived vesicles share similar pathways of cellular exit and entry has not escaped attention, and this topic has been extensively reviewed (van Dongen et al., 2016). Classically, viruses can evade the immune response by mutating their surface proteins or by inducing immunosuppressive changes within specific cell types (Hashimoto et al., 2009; Ramakrishnaiah et al., 2013; Sun et al., 2014). Recent studies reveal that key viral proteins and/or nucleic acids are secreted from infected cells within host-derived vesicles with corresponding host surface proteins (Robinson et al., 2014), thus facilitating immune evasion while retaining their infectious capacity. Other reports, however, demonstrate that viral species ejected within host derived MVs are internalized more efficiently by dendritic cells (Feng et al., 2015), and this may represent an adaptation of the infected cell to facilitate immune capture of viral particles attempting to infect other cells. As consideration is given to the development of vesicle-based therapeutics, understanding mechanisms involved in cargo sorting in various contexts is imperative in order to deduce which scenarios reflect viral attempts at immune evasion and which are sophisticated host-derived immune defense mechanisms.

## EVs as biomarkers

As readily measurable indicators of a disease process or its progression, biomarkers can facilitate the ability of clinicians to diagnose, prognosticate, and measure responses to therapy. While tissue biopsy sampling represents the gold standard in providing relevant histopathologic or molecular diagnostic information, such approaches are invasive and subject to risk, can yield false negative results, and provide only a single snapshot in time. The concept of a “liquid biopsy” is gaining favor (Karachaliou et al., 2015) as a means to overcome such limitations, with EVs within relevant body fluids emerging as leading contenders to provide a lens onto dynamic internal pathophysiologic processes such as immune disorders. Decreased (as in smoking) or increased (as in infection) levels of vesicular SOCS in lung lavage fluid may provide useful information about lung immune status, but their utility as biomarkers will require them being readily measured by more accessible means, such as plasma, exhaled breath condensate, or saliva.

## Conclusions

Herein we have reviewed our recently published data on transcellular delivery of SOCS proteins from AMs to AECs as a means of restraining inflammation on the pulmonary alveolar surface (Bourdonnay et al., 2015; Speth et al., 2016). We have used our findings regarding modulation of SOCS packaging within EVs and of EV uptake by relevant microenvironmental determinants to illustrate how aspects of vesicle trafficking and cargo delivery can be regulated in a dynamic manner, and to highlight significant gaps in current knowledge. Our own studies have focused on the novel role of SOCS proteins, but the effects of environmental cues on the global content and delivery of vesicular protein, lipid, and nucleic acid cargo, of course, remains to be elucidated.

## Author contributions

All authors listed, have made substantial, direct and intellectual contribution to the work, and approved it for publication.

## Funding

This work is supported by NIH R01 HL-125555 (to MP-G). JMS was supported by NIH T32 HL-774923.

### Conflict of interest statement

The authors declare that the research was conducted in the absence of any commercial or financial relationships that could be construed as a potential conflict of interest. The handling Editor declared a shared affiliation, though no other collaboration, with the authors and states that the process nevertheless met the standards of a fair and objective review.
